# Adhesion to Carbon Nanotube Conductive Scaffolds Forces Action-Potential Appearance in Immature Rat Spinal Neurons

**DOI:** 10.1371/journal.pone.0073621

**Published:** 2013-08-12

**Authors:** Alessandra Fabbro, Antonietta Sucapane, Francesca Maria Toma, Enrica Calura, Lisa Rizzetto, Claudia Carrieri, Paola Roncaglia, Valentina Martinelli, Denis Scaini, Lara Masten, Antonio Turco, Stefano Gustincich, Maurizio Prato, Laura Ballerini

**Affiliations:** 1 Life Science Department, University of Trieste, Trieste, Italy; 2 Department of Chemical and Pharmaceutical Sciences, University of Trieste, Trieste, Italy; 3 Department of Biology, University of Padua, Padova, Italy; 4 Department of Neuroscience, Psychology, Drug Research and Child's Health, University of Florence, Florence, Italy; 5 Innovation and Research Center, Fondazione Edmund Mach, San Michele all’Adige, Trento, Italy; 6 European Molecular Biology Laboratory, Mouse Biology Unit, Monterotondo (Rome), Italy; 7 International School for Advanced Studies (SISSA), Trieste, Italy; 8 European Bioinformatics Institute (EMBL-EBI), Hinxton, United Kingdom; 9 International Centre for Genetic Engineering and Biotechnology (ICGEB), Trieste, Italy; 10 SENIL, ELETTRA Synchrotron Light Source, Trieste, Italy; Institute for Frontier Medical Sciences, Kyoto University, Japan

## Abstract

In the last decade, carbon nanotube growth substrates have been used to investigate neurons and neuronal networks formation in vitro when guided by artificial nano-scaled cues. Besides, nanotube-based interfaces are being developed, such as prosthesis for monitoring brain activity. We recently described how carbon nanotube substrates alter the electrophysiological and synaptic responses of hippocampal neurons in culture. This observation highlighted the exceptional ability of this material in interfering with nerve tissue growth. Here we test the hypothesis that carbon nanotube scaffolds promote the development of immature neurons isolated from the neonatal rat spinal cord, and maintained in vitro. To address this issue we performed electrophysiological studies associated to gene expression analysis. Our results indicate that spinal neurons plated on electro-conductive carbon nanotubes show a facilitated development. Spinal neurons anticipate the expression of functional markers of maturation, such as the generation of voltage dependent currents or action potentials. These changes are accompanied by a selective modulation of gene expression, involving neuronal and non-neuronal components. Our microarray experiments suggest that carbon nanotube platforms trigger reparative activities involving microglia, in the absence of reactive gliosis. Hence, future tissue scaffolds blended with conductive nanotubes may be exploited to promote cell differentiation and reparative pathways in neural regeneration strategies.

## Introduction

Nanomaterials are increasingly used for organ engineering purposes [1,2]. Scaffolds with manufactured three-dimensional properties may promote cells reorganization into functional tissue. This possibility has driven a growing interest in studying physical-chemical features of scaffolds at the nano-scale, to activate cell-specific molecular machineries [1–4].

Scaffolds blended with various materials have been constructed for the repair of different tissues, such as bones, liver and other organs [1,2,5–7]. However, attempts to construct scaffolds for the repair of the central nervous system (CNS) have had limited success, because of its intrinsic complexity, low regenerative potential and anatomically restrictive nature, which pose a unique set of challenges [8–13]. Despite this fact, an increasing amount of studies in modern neuroscience addresses the ability of growth substrate topography or physical features in driving neuronal networks reconstruction. In cultured systems, the interaction of neurons with their growth substrate may influence neuron differentiation, morphology, adhesion and outgrowth [13–19].

Our approach to address this issue was to incorporate neuronal cultures to artificial conductive nanostructures, namely carbon nanotubes. Recently, carbon nanotubes have attracted tremendous attention for the development of nano-bio hybrid systems able to govern cell-specific behaviors in cultured neuronal networks and explants [20–27] and have been shown to promote proliferation of neonatal cardiac myocytes [28].

Carbon nanotubes are cylindrically shaped nanostructures, made of one or more concentric rolled-up graphene sheets, which possess peculiar properties including high surface area, high mechanical strength, ultra-light weight, rich electronic properties, and excellent chemical and thermal stability [29,30]. *In vivo*, carbon nanotubes have been shown to be a blood compatible and a suitable scaffold for bone regeneration [31,32] or, *in vitro*, for cultured synaptic network formation [22–24,26,27,33] and neonatal cardiomyocyte maturation [28].

In the present work we investigate the interaction between carbon nanotube scaffolds and immature spinal cord neurons. Here we show that *in vitro* spinal neurons adherent to carbon nanotube substrates undergo a functional maturation characterized by an earlier appearance of voltage dependent currents and of action potentials. To address the mechanistic pathways between the enhanced membrane excitability profile of spinal neurons and their contact to carbon nanotube scaffolds, we perform electrophysiological studies associated, for the first time, to gene expression analysis. Carbon nanotube substrates induce neuronal modifications specific to spinal immature neurons, and ultimately modulate their electrogenic development.

## Materials and Methods

### Ethics Statement

All work on animals (neonatal rats) was done according to the EU guidelines (86/609/CE) and the current Italian law (decree 116/92). The study was approved by the Italian Ministry of Health, in agreement with the EU Recommendation 2007/526/CE. Animals were hosted by the University of Trieste Animal Facility (Department of Life Sciences), Italy, authorized by the Italian Ministry of Health, and breeding conditions and procedures complied with EU guidelines (86/609/CE) and Italian law (decree 116/92). Neonatal animals were sacrificed by rapid decapitation and the tissue of interest (spinal cord) harvested, all efforts were made to minimize suffering. The work has been performed on the explanted tissue and did not require ethical approval, as stated by the current Italian law (decree 116/92). The entire procedure is in accordance with the regulations of the Italian Animal Welfare Act, with the relevant EU legislation and guidelines on the ethical use of animals and is approved by the local Authority Veterinary Service.

### Carbon nanotube substrates and culture preparation

Stable and homogeneous growth substrates were obtained by multiwalled carbon nanotubes (MWCNT) of 20-30 nm diameter (Nanostructured & Amorphous Materials, Inc.) used as received and functionalized using the 1,3 dipolar cycloaddition of azomethine ylides [22]. The reaction generates pyrrolidine groups on the entire nanotube surface, which increase notably MWCNT solubility in organic solvents. Functionalized MWCNTs were dissolved in dimethylformamide (0.01 mg/mL) or ethyl acetate (0.1 mg/mL) and the proper amount of the solution was deposited by drop casting (dimethylformamide solution) or sprayed (ethyl acetate solution) on glass coverslips placed on a hot plate at 100 °C, achieving a density of the MWCNT film over the glass of about 7x10^-5^ mg/mm^2^. Then, layered coverslips were placed in an oven at 350 °C under N_2_ atmosphere for 20 min, a procedure leading to MWCNT de-functionalization. To assess MWCNT degree of purity and de-functionalization, TGA-Q500 (TA Instruments) was used to record thermo-gravimetric analysis (TGA) under N_2_ or under air, by equilibrating at 100 °C, and following a ramp of 10 °C/min up to 1000 °C. De-functionalized MWCNTs deposited on glass showed less than 10% metal content [34]. TGA was routinely repeated to test every new MWCNT batch. MWCNTs thin film was characterized by sheet resistance measurements obtained using a Jandel four tips probe linked to Jandel RM3000 multimeter. The calculated value of conductivity for the type of MWCNTs network used in this study is 6.25 S/cm. Control substrates were prepared by coating glass coverslips with polyornithine (0.003 mg/mL).

Dissociated neuronal cultures were prepared from neonatal Wistar rat spinal cords at postnatal day (P) P1-P4. The vertebral columns were exposed dorsally and the spinal cords were removed in an ice-cold phosphate buffered saline (PBS). The tissue was grossly crumbled, digested with papain (1 mg/mL; Sigma) in EBSS (Gibco; bubbled with 95% oxygen 5% CO_2_) for 45 min and then washed with EBSS plus 1 mg/mL bovine serum albumin (BSA, Sigma). The tissue was re-suspended in 1 mL of EBSS plus 1 mg/mL BSA and 0.01% DNAse (Sigma). Cells were gently mechanically dissociated and the gross tissue was allowed to deposit, while the supernatant was collected. After repeating this procedure 3 times, 5 mL of EBSS plus 10 mg/mL BSA were added to the collected supernatant. Upon centrifugation (6 min at 600 rpm) the supernatant was then removed and cells were re-suspended in culture medium [Eagle’s minimal essential medium plus Glutamax, (Gibco), supplemented with 20 mM glucose, 100 U/ml penicillin, 0.1 mg/ml streptomycin, 5% horse serum and 5 ng/mL Nerve Growth Factor (NGF, Alomone Labs)]. A drop of the cell suspension was delivered on a coverslip (control or MWCNT coated glass coverslips), cells were allowed to attach to the substrate for 1 h at 37 °C and 2 mL of culture medium were finally added. Cells were maintained at 37° C in a humidified incubator with 5% CO_2_ atmosphere and used for the experiments after 8 days of *in vitro* growth (if not otherwise indicated). In both carbon nanotube and control culturing conditions 5 ng/mL of nerve growth factor was added to the culture medium [35–37]. We used a low NGF concentration reminiscent to that found in the cerebrospinal fluid of newborn rats [38], to mimic such condition. This is more suited to unmask any potential impact of MWCNT substrates on immature spinal neurons.

Neuronal density was measured by means of immunocytochemistry experiments (see below), quantifying β-tubulin III-positive cells in randomly chosen visual fields and normalizing the measured cell number for the visual field area.

### Electrophysiological recordings

For each experiment, a coverslip with the spinal culture was mounted in a recording chamber on an inverted microscope and perfused with a recording solution containing (in mM): NaCl 150, KCl 4, MgCl_2_ 1, CaCl_2_ 2, HEPES 10, glucose 10, pH 7.4 with NaOH. Whole-cell patch-clamp experiments were performed using patch pipettes (4-7 MΩ) filled with (in mM): K-gluconate 110, KCl 30, HEPES 10, GTP 0.3 and MgATP 4, pH adjusted to 7.35 with KOH. Electrophysiological recordings were acquired using a Multiclamp 700B amplifier (Molecular Devices), sampled at 10 kHz and digitized by a Digidata 1440A analog-to-digital converter. Bridge balance (in the current clamp mode) and series resistance (in the voltage clamp mode) were monitored throughout the experiment; neuronal passive properties were routinely measured in voltage clamp mode. Data were analyzed using the pCLAMP software (Molecular Devices). All experiments were performed at room temperature.

### Immunofluorescence and electron microscopy

For β-tubulin III immunofluorescence experiments, dissociated spinal cord cultures were fixed with paraformaldehyde (PFA, 4% in PBS, Sigma). Coverslips were rinsed with PBS, incubated for 30 minutes in a blocking solution (5% BSA -Sigma-, 0.3% Triton X-100 -Carlo Erba-, 1% Fetal Bovine Serum –Gibco- in PBS), then incubated with anti-β-tubulin III primary antibody (rabbit polyclonal; 1:250, Sigma) overnight at 4° C. The samples were then washed three times with PBS and incubated with the secondary antibody (Alexa Fluor-594 goat anti-rabbit, 1:300, Invitrogen) for 2 hours at room temperature (RT). Finally, the coverslips were washed three times in PBS and mounted in glycerol plus DABCO (2.5% w/v).

Immunofluorescence experiments for Iba1 and GFAP were performed on PFA-fixed samples (see above). Coverslips were incubated for 1 hour in a blocking solution (5% BSA -Sigma-, 0.1% Triton X-100 -Carlo Erba-, 5% Fetal Bovine Serum –Gibco- in PBS), and then incubated with the primary antibodies (Iba1: rabbit polyclonal, Wako, 1:1000; GFAP: mouse monoclonal, Sigma, 1:200) for 1 hour at RT. The samples were washed with PBS and incubated with the secondary antibodies (Alexa Fluor-594 anti-rabbit, 1:500; Alexa Fluor-488 anti-mouse, 1:250; Invitrogen) for 1 hour at RT. The coverslips were finally washed in PBS and mounted.

For Alox15 immunofluorescence experiments, the samples were washed with 0.1 M cacodylate buffer (pH = 7.2), fixed with a solution containing 2% glutaraldehyde (Fluka, Italy) in 0.1 M cacodylate buffer for 1 h at RT and washed three times with 0.1 M cacodylate buffer. The samples were then treated to block any non-specific binding with 1% BSA (Bovine serum Albumin, SIGMA) for ≥1 hour and finally incubated for 2 hours at room temperature with the rabbit polyclonal anti-LO (H-235), 1:50 (sc-32940, Santa Cruz Biotechnology, Inc) diluted in PBS plus 0.1% BSA and 0.05% Tween-20. All washing steps and secondary antibody dilution were performed with PBS plus 0.1% BSA. The incubation with the secondary antibody (goat anti-rabbit conjugated to Alexa Fluor-594; 1:1000; Invitrogen) was performed for 1 hour at room temperature. Finally, coverslips were mounted in Vectashield with DAPI to counterstain the nuclei (VECTOR Lab Inc, Burlington, USA).

Images from immunofluorescence experiments were obtained with both a conventional fluorescence microscope (Leika) and with a Zeiss LSM 510 META Confocal Microscope (Zeiss, Germany; 63X oil immersion objective).

Scanning electron microscopy (SEM) images were acquired collecting backscattered and secondary electrons on a commercial SEM (Gemini SUPRA 40, Carl Zeiss NTS GmbH, Oberkochen). Cultures grown on MWCNT carpets were fixed with 2% glutaraldehyde dissolved in cacodylate buffer (0.1 M, pH 7.2) in the dark for 1 hour at room temperature. After fixation samples were carefully rinsed with cacodylate buffer, dehydrated in absolute ethanol and stored before use in a nitrogen box. In order to prevent electron induced surface charging, low accelerating voltages (0.8÷1.5 keV) were used for cells visualization. Samples were imaged without any prior metallization process.

### Microarrays

Microarray experiments were performed on three different biological replicates (three culture series) for each of the two culturing conditions (control and MWCNT substrates). Total RNA was isolated and DNAse treated with Absolutely RNA Nanoprep kit (Stratagene) according to manufacturer’s instructions. RNA quality was assessed using an Agilent 2100 Bioanalyzer (Agilent Technologies). RNA was quantified with a NanoDrop 1000 spectrophotometer (Thermo Scientific). A 100 ng-amount of each total RNA sample was then amplified and labeled with the Illumina RNA amplification kit according to manufacturer’s instructions.

Labeled cRNA was hybridized on Affymetrix Rat Genome 230 2.0 Arrays. Hybridized arrays were stained and washed (GeneChip Fluidics Station 450), then scanned with the GeneChip Scanner 3000 7G. Cell intensity values and probe detection calls were computed using the AffymetrixGeneChip Operating Software (GCOS).

### Western blot

For protein analysis, samples were lysed in 5X SDS-PAGE sample buffer (0,225 M Tris-HCl, pH 6.8, 50% Glycerol, 5% SDS 10%, 0.05% Bromophenol blue, and a Protease Inhibitor Cocktail -Sigma). Equal amounts of protein were resolved on SDS-PAGE minigels and transferred to PVDF membranes (GE Healthcare). Immunoblots were blocked for at least 1 hour at 37 °C, in a solution of 10% nonfat dry milk in PBS with 0.05% Tween-20. Membranes were incubated with primary antibodies (see below) for 2 hours at room temperature in 5% PBS with 0.05% Tween-20, and washed in PBS, 0.05% Tween-20 with gentle mixing. Secondary antibodies were diluted in 5% of PBS with 0.05% Tween-20, and incubated with the membranes for 45 min at room temperature. Proteins were detected by enhanced chemiluminescence (GE Healthcare).

The primary antibodies used were as follows: rabbit polyclonal anti-Robo1 (H-200), 1:250 (sc-25672, Santa Cruz Biotechnology, Inc); rabbit polyclonal anti-lipoxygenases (H-235), 1:200 (sc-32940, Santa Cruz Biotechnology, Inc); mouse monoclonal anti-paxillin, 1:200 (sc-373880, Santa Cruz Biotechnology, Inc), and mouse monoclonal anti-Actin (C-2), 1:10000 (sc-8432, Santa Cruz Biotechnology, Inc). The secondary antibodies used were as follows: HRP-conjugated anti-rabbit, 1:2000 (P0448, DAKO), and HRP-conjugated anti-mouse, 1:2000 (P0161, DAKO).

Band densities of Alox15 and Robo1 proteins were normalized to each actin internal control.

### RNA/DNA Isolation and quantitative RT-PCR (qPCR)

Total RNA and DNA were purified from dissociated spinal cultures at 3-5 days in vitro (DIV), 7-8 DIV, and 9-10 DIV (named 4 DIV, 8 DIV and 10 DIV, respectively), isolated using the Trizol-method (Invitrogen), and quantified by spectrophotometry. cDNA was prepared from 1 µg total RNA by reverse transcription with MMLV-RT (Invitrogen), using random hexamer primers (Invitrogen) following standard protocols (Invitrogen). RNA expression levels for Alox15 and Robo1 were quantified with real-time TaqMan reverse transcriptase (RT)-PCR using C1000 CFX96 Real-Time System (Bio-Rad). TaqMan reactions were carried out in 96-well plates using cDNA, Taqman universal PCR master mix, predesigned and preoptimized TaqMan. Gene expression assays, including specific primers and fluorescent probes (Alox15 Rn00578745_g1 FAM; Robo1 Rn00573395_m1 FAM; glyceraldehyde-3-phosphate dehydrogenase –GAPDH- Rn999999_s1 FAM), and water to a final volume of 50 µL, were performed according to manufacturer’s instructions. GAPDH mRNA was used as an endogenous control. Primer pairs for Alox15 and Robo1 were obtained from the Applied Biosystems catalogue for quantitative gene expression analysis of the genes of interest. RT and template controls were used to monitor any possible contaminating amplification according to manufacturer (Applied Biosystems). The temperature protocol was the following: 3 min at 95 °C,10 min at 95 °C, and 30 min at 60 °C, followed by 39 cycles of 10 s at 95 °C and 30 s at 60 °C. GAPDH expression was similar in all study groups and was therefore employed to normalize for differences in RNA quantity and RT-efficiency.

### Statistical analysis

Results are presented as mean ± S.E.M.; n is the number of neurons, if not otherwise indicated. Statistically significant difference between two datasets was assessed by Student’s t test (after validation of variances homogeneity by Levene’s test) for parametric data and by Mann-Whitney for non-parametric ones. The comparison between data distributions was made by the Kolmogorov-Smirnov test.

For microarray experiments, expression analysis of Affymetrix CEL files has been performed using EntrezGene Custom CDF annotation files proposed by Dai et al. [39], then RMA algorithms [40] for normalization procedures was applied. Differentially expressed genes were calculated by using the Rank Product algorithm [41]. Each sample was considered a separate origin. To ensure statistical significance, the maximum cut-off for the percentage of false positives (pfp) was set at 0.05. Class enrichment (with respect to the entire platform) has been tested with the hypergeometric distribution using KEGG pathways [42] and Gene Ontology categories [43]. Statistical significance was determined using a False Discovery Rate (FDR) minor or equal to 0.05. All the annotations procedures and statistical analyses were performed with R software (http://www.r-project.org) using the BioConductor suite.

Microarray data reported in this work have been submitted to the GEO (Gene Expression Omnibus) database with accession number GSE27212.

## Results and Discussion

**Figure 1:**
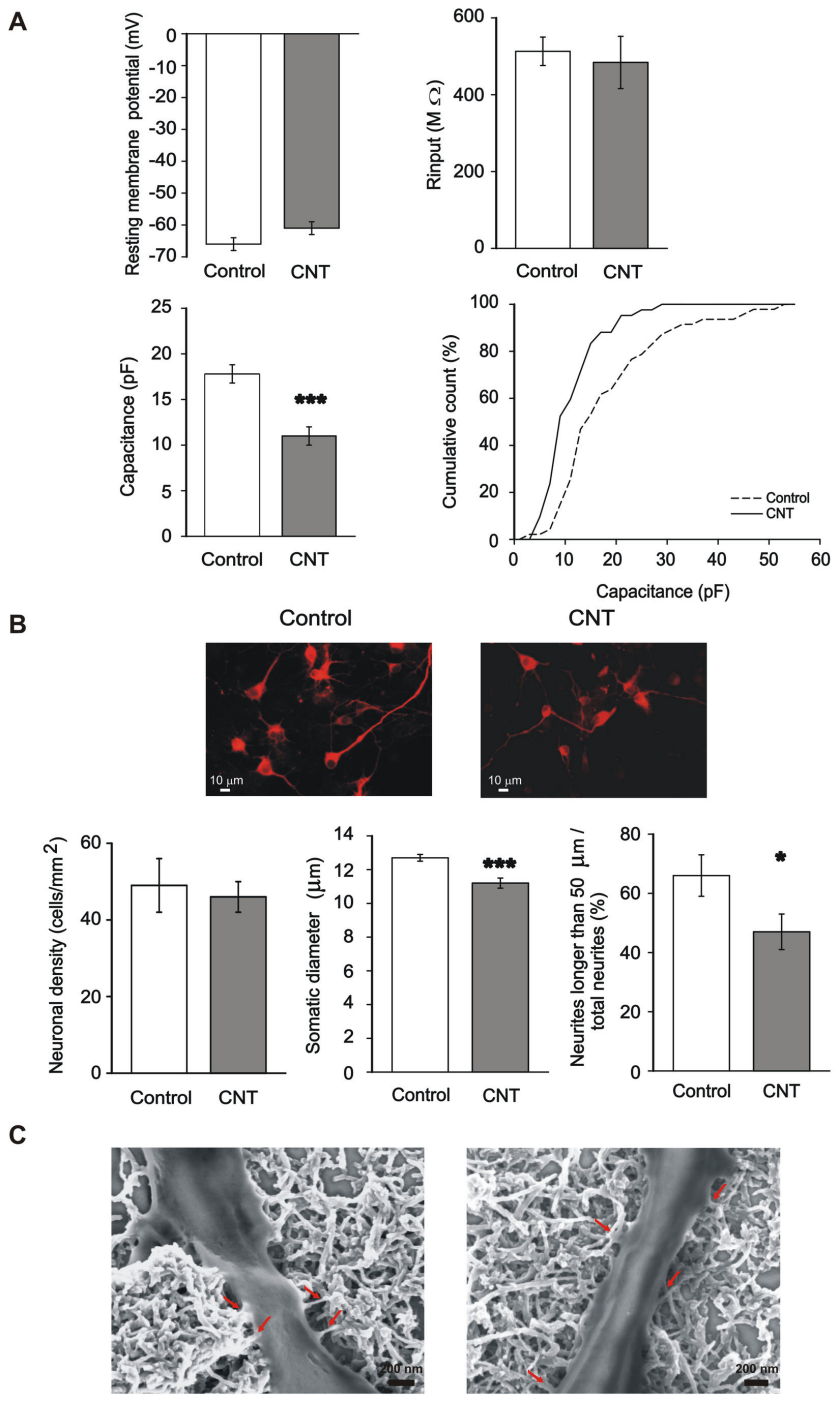
Carbon nanotubes substrate selectively affects membrane properties and neurite extension of spinal neurons. (A) Spinal neurons grown on MWCNT show similar resting potential and membrane resistance (R_input_) with respect to control neurons (top), while membrane capacitance, indicative of total cell membrane extension, is significantly lower in CNT neurons (bottom left, ***: P<0.001; bottom right, cumulative distribution, KS test: P<0.01). (B) Top, typical examples of spinal cord neurons cultured in control condition or on MWCNTs, labeled with the antibody against the neuronal marker β-tubulin III to visualize neuronal morphology. Bottom, despite a similar neuronal density (left), CNT cultures showed a slightly, but significantly lower somatic neuronal diameter compared to control cultures (middle; ***: P<0.001), together with a lower number of long neurites (right; *: P<0.05). These findings are in agreement with the lower membrane capacitance values found in CNT neurons. (C) SEM images of neurites from spinal neurons grown on a MWCNT layer, showing the numerous and very tight contacts between MWCNTs and neuronal membranes (red arrows).

In our recent work we developed and characterized artificial nanomaterial-based scaffolds to explore the ability of conductive carbon nanotube substrates to interface neurons and influence their performance and efficacy in building synaptic networks in culture [22–24,26]. Here we tested how MWCNTs supports may alter immature spinal neuron ability to perform action potentials along with *in vitro* cell development. We examined and compared neonatal rat spinal neurons cultured on homogeneous and stable layers of MWCNTs [23,24,26] (Figure 1C) with those grown on control substrates (named CNT and control, respectively). CNT dissociated spinal neurons were able to adhere, grow and survive while extending several neuritis on the growth substrates, confirming the general biocompatibility of MWCNT scaffolds reported for different neuronal [20,22–26,33,44] and non-neuronal [28] excitable cells maintained in culture.

### Carbon nanotube scaffolds force mature neuronal excitability

In a first set of experiments (n=10 culture series) the membrane passive properties of spinal neurons grown integrated to MWCNT were measured and compared to those of control cells. Histograms in Figure 1A summarize these results: recorded neurons displayed similar and homogeneous membrane resting potential and input resistance values (top plots), indicative of a healthy cell population in both culturing conditions. Conversely, when analyzing membrane capacitance, CNT neurons displayed a 37% reduction in this parameter (from 17.9 ± 1.5 pF in control to 11.2 ± 0.9 pF on MWCNT; Figure 1A, bottom plots), as shown in the cumulative plot of Figure 1A (KS test: P<0.01). Cell capacitance is an indirect estimate of membrane surface and the values measured in neurons belonging to the two culturing conditions might suggest a difference in their soma size and/or in the extension of their neurites. To test this hypothesis, we visualized neuronal morphology by immunofluorescence experiments with an antibody recognizing the neuron-specific marker β-tubulin III. In both control and CNT cultures we documented a comparable density of spinal neurons after one week of *in vitro* growth (Figure 1B, n=33 visual fields, n= 3 cultures in control; n=34 visual fields, n=3 cultures on MWCNT) and we directly measured the somatic neuronal diameter. CNT neurons displayed on average a 12% reduction in diameter (from 12.7 ± 0.2 µm, n=89 control to 11.2 ± 0.3 µm, n=43, CNT neurons; P<0.001; Figure 1B) when compared to control neurons. This small, although significant, difference in cell diameter may account for the detected decrease in cell capacitance observed in CNT neurons. In fact, in a gross approximation, the cell body shape can be assimilated to a sphere, thus a 12% difference in diameter accounts for a 23% difference in the surface area (reflected, in our case, by cell capacitance). We further measured neurite extension by quantifying the presence of long neuritis (>50 µm) over the total number of neurites per cell. While the number of total neurites per cell was the same in control and CNT (4 ± 0.3 and 3.8 ± 0.3, n=23 in control and n=27 in CNT, respectively), there was a 29% decrease in the number of long neurites in neurons grown attached to MWCNT substrates (from 66 ± 7%, n=23 in control to 47 ± 6%, n=27 in CNT; P<0.05; Figure 1B) which may also contribute to the smaller capacitance values measured in CNT neurons.

This result is surprising, given that in other culture systems an increase in axonal growth was detected when neurons were interfaced to MWCNTs [25,26]. To note is that in our previous experiments [26] we measured SMI32 positive neurite outgrowing from dorsal root ganglia or spinal ventral horns, emerging from ganglion cells and motoneurons, developed in organotypic cultures [26]. Conversely, here we are testing isolated small (as suggested by the average soma diameter) spinal interneurons in low growth factor culturing conditions. We further checked whether the adhesion of neuronal processes to the MWCNT substrate was comparable to what reported in previous experimental settings [23,26,33]. To this aim a set of cultures was analyzed by SEM, as shown in the sample micrographs of Figure 1C. In all these measures (n=3 cultures) we always observed the typical tight and intimate contacts (red arrows in Figure 1C) between small neurites and MWCNT, indicative of a strong membrane adhesion to these substrates (see also Figure S1).

**Figure 2:**
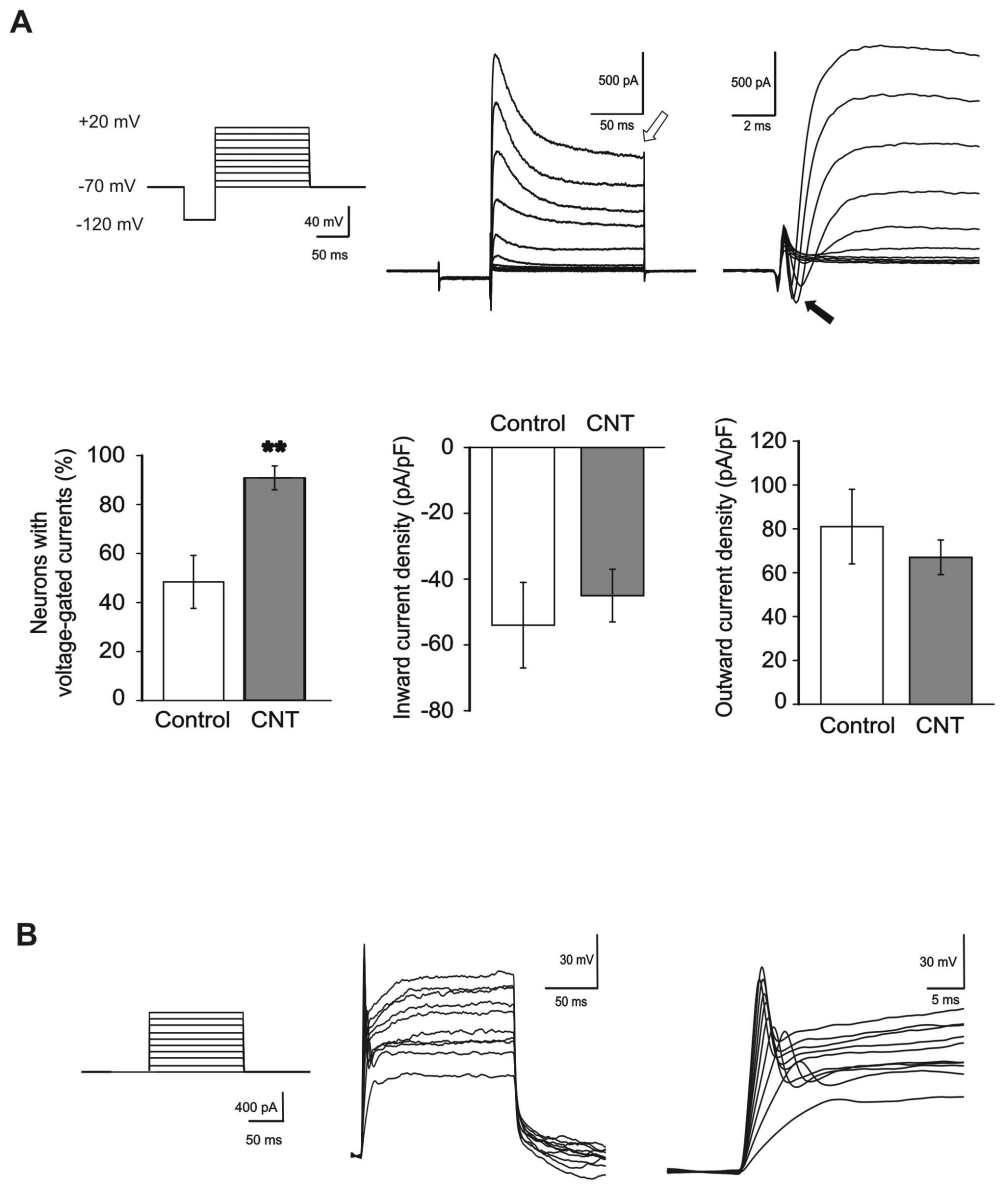
MWCNTs boost the functional maturation of spinal neurons. (A) Top left, voltage-clamp stimulation protocol to test the presence of voltage-dependent currents. Top middle (and right, in extended time scale), typical recordings from a spinal neuron displaying voltage-dependent currents. Note the presence of both outward (open arrow, middle panel; K^+^) and inward (filled arrow, right panel; Na^+^) voltage-dependent currents. The fraction of neurons displaying voltage-dependent currents is considerably higher in CNT neurons with respect to controls (bottom left; **: P<0.01), while current density is similar for inward and outward currents in both culturing conditions (bottom middle and right). (B) Left, current-clamp stimulation protocol to test the neuronal ability to generate action potentials. Middle (and right, in extended time scale), example of action potentials generated by a spinal neuron.

We next addressed the impact of growth substrates in the maturation of spinal interneuron excitability after 8 days of *in vitro* growth, thus, in this culturing condition, before the appearance of any detectable synaptic activity. We measured, under voltage clamp mode, the expression of functional voltage gated ion channels. Traditionally, the increasing expression of voltage dependent conductance is an accepted index of neuronal development and differentiation beyond positivity to β-tubulin III and towards the mature, electrically active, neuronal phenotype [45–49]. Whole-cell currents were recorded from neurons held at -70 mV of membrane potential. We applied 10 consecutive square (duration 150 ms) depolarizing steps of increasing amplitude (10 mV increments) to elicit voltage-dependent currents, preceded by a 50 ms hyperpolarization at -120 mV holding potential, to remove residual Na^+^ channels inactivation (Figure 2A). This experimental protocol is effective in inducing inward currents due to the opening of Na^+^ and Ca^2+^ voltage activated channels and outward currents due to the opening of K^+^ voltage activated channels in neurons [50]. We focused our attention on two components of each response: the steady state outward K^+^ currents (Figure 2A, top middle, white arrow) and fast inward currents generated by Na^+^ voltage-dependent channels (Figure 2A, top right, black arrow, note the different time scale). These two current components are crucial mechanisms for action potential generation [51,52]. We identified outward and inward currents as K^+^ and Na^+^ mediated currents, respectively, by using selective and specific K^+^ and Na^+^ channel blockers (tetraethylammonium –TEA 10 mM- and tetrodotoxin -TTX 1 µM-, respectively; n=3 not shown). CNT spinal cultures showed a strong increase (87%) in the probability of finding neurons displaying voltage-gated currents (from 46 ± 10%, n=56 control neurons to 86 ± 5%, n=46 CNT neurons; P<0.01; plot in Figure 2A). We compared the current density recorded in a subset of control and CNT neurons expressing inward and outward components by normalizing the current amplitude obtained at 0 mV depolarization to the cell capacitance. These values did not differ between the two culture groups (plots in Figure 2A for inward and outward currents; n=16 control and n=18 in CNT). This finding suggests that control and CNT neurons, once reached a degree of differentiation where voltage dependent measurable K^+^ and Na^+^ currents were expressed, displayed comparable channel density. However, in the two culture systems, the amount of neurons able to display such membrane properties is highly different.

The presence of Na^+^ and K^+^ currents predicts the ability of spinal neurons to generate action potentials. We tested this prediction by switching the recording from voltage to current-clamp mode after assessing the presence of voltage dependent currents. We injected in these neurons current square pulses (150 ms) of increasing amplitude (in 100 pA increments; Figure 2B) and we monitored the membrane potential changes. We confirmed that neurons, which showed voltage-activated currents, were able to generate action potentials (Figure 2B). Action potentials displayed on average 98 ± 3 mV of amplitude (n=11).

Thus we show that small interneurons grown in contact to conductive MWCNT anticipate the expression of functional markers of maturation, such as the ability to generate voltage dependent currents and action potentials. We believe that this is a genuine phenomenon, in fact previous reports in analogous recording conditions ruled out the possibility that similar or even higher capacitance values in control neurons might limit space-clamp significantly, impairing the detection of action potential or voltage activated currents [22,53–55]. MWCNT growth supports were reported to affect various aspects of excitable cell function in culture [22–24,26,28]. Here, cell adhesion to MWCNT drives the functional maturation of spinal neurons, forcing towards neuronal differentiation regardless the appearance of functional synaptic contacts.

### Carbon nanotubes affect spinal cell gene expression

Since biophysical measures suggested that spinal interneurons grown on MWCNT exhibit a more mature electrophysiological phenotype, we were interested in assessing whether and how this might be related to changes in gene expression.

**Figure 3:**
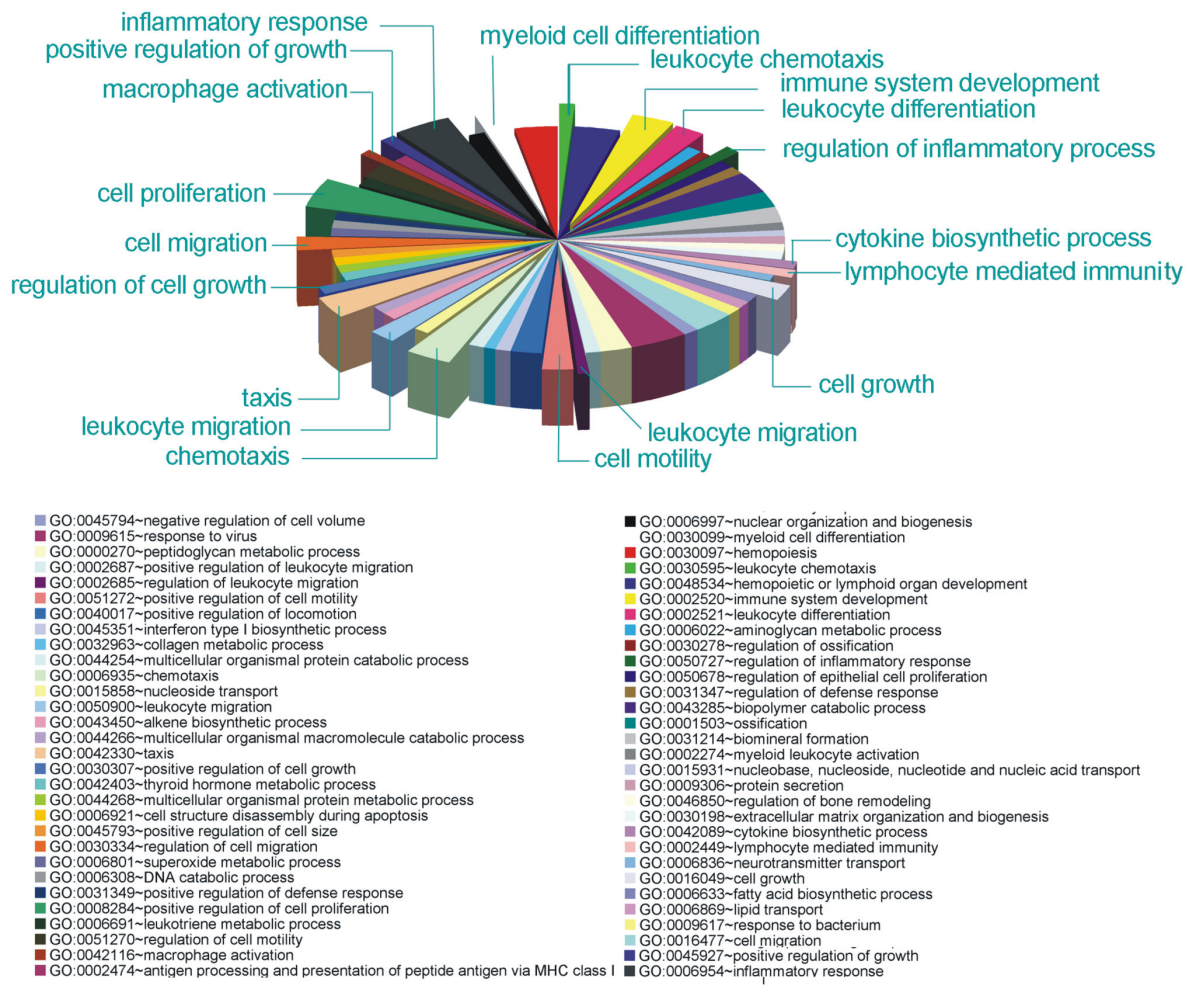
Gene Ontology enrichment analysis showing gene categories whose expression is modulated by MWCNTs. See Table S2 for statistics.

To this purpose, at day 8 after plating, we performed mRNA profiling experiments to investigate the gene expression level in CNT and in control cultures by means of microarray platforms. As a general observation, the gene profile of cells cultured on MWCNT was very similar to that depicted in control cells. Differentially expressed genes (DEGs) analysis identified a total of 5 down-regulated and 46 up-regulated genes in CNT cultures, when compared to controls (with a fold change (FC) > 2 and FC<0.5, corrected p-value < 0.05, in Table S1). To elucidate the functional properties of genes which expression changed when cultures were developed on MWCNT supports, we performed Gene Ontology enrichment analysis and we focused on significantly affected biological processes. By this procedure we identified process categories in which gene expression resulted differently modulated by MWCNT substrates. Among the identified genes, several have been described as involved in cell communication (FDR = 0.006), taxis and chemotaxis (FDR = 0.005 and 0.006, respectively), cell growth and differentiation (FDR = 0.006, 0.047 and 0.018, respectively) as well as immune-related functions (FDR=0.022) and inflammation (FDR=0.023; Figure 3; Table S2).

**Figure 4:**
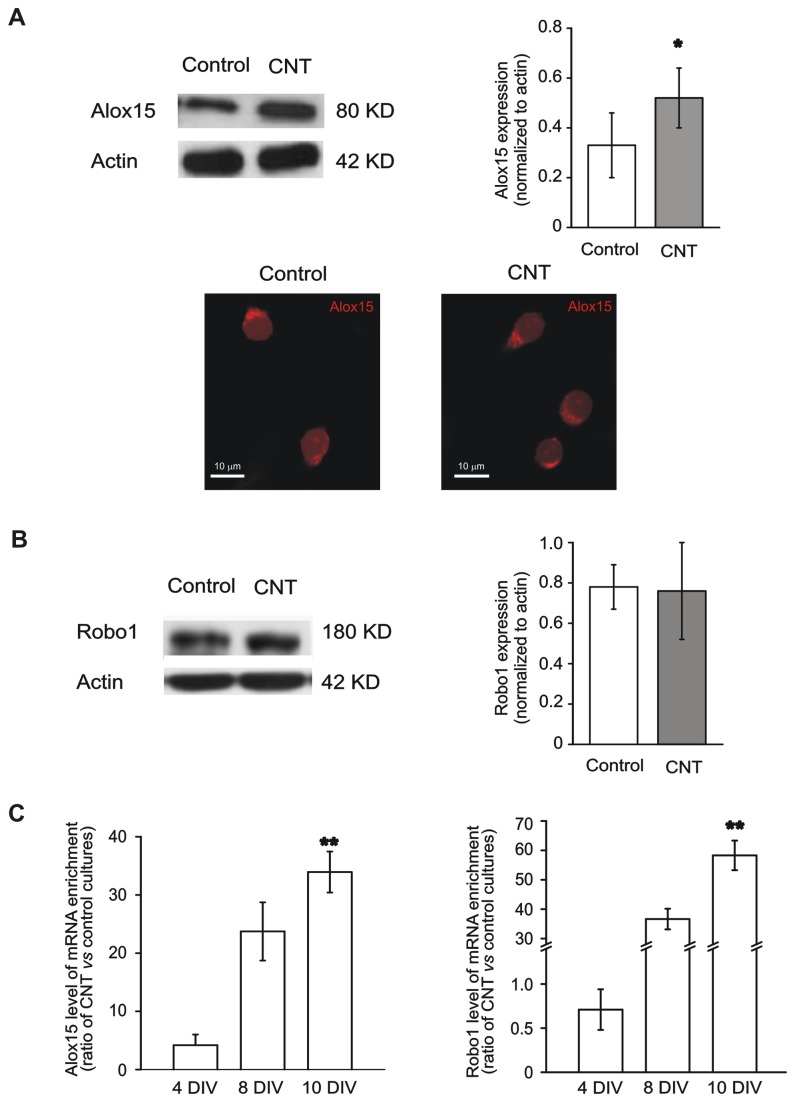
The expression of *Alox15* and *Robo1* is increased by MWCNT substrate. (A) Top left, Western blot analysis of the Alox15 protein (migrated at approximately 80 KD) isolated from control (left lane) and CNT (right lane) spinal cultures, showing the strong increase in Alox15 expression in CNT cultures. Top right, quantification of the Alox15 protein level normalized against actin. Bottom, confocal images of control and CNT neurons, stained using the anti-Alox15 antibody. Note the punctate appearance of strongly Alox15-immunopositive cytoplasmatic structures in both culturing conditions. (B) Left, Western blot analysis of the Robo1 protein (migrated at approximately 180 KD) from control (left lane) and CNT (right lane) cultures. Robo1 expression level is unaffected by the MWCNT substrate (right). (C) Time course of *Alox15* (left) and *Robo1* (right) expression levels by transcript-specific real time PCR in CNT cultures normalized to control. The expression of both proteins is progressively upregulated in CNT cultures during *in vitro* development.

It was particularly noteworthy that, among those genes whose expression was significantly modulated by MWCNT supports, some are potentially involved in neuronal maturation. *Nedd4* (neural precursor cell-expressed developmentally down-regulated gene 4) encodes for an ubiquitin ligase involved in the degradation of cellular proteins, including Na^+^ voltage-activated channels, and in the negative modulation of neuronal Na^+^ channels activity [56]: its down-regulation in CNT cultures is thus consistent with the presence of a significantly larger neuronal population able to generate action potential (i.e., expressing functional Na^+^ voltage-gated channels). On the other hand, some up-regulated genes also raised particular interest being traditionally involved in neuronal maturation and physiology. In particular *Alox15* and *Robo1* captured our attention. The product of the *Alox15* (arachidonate 15-lipoxygenase) gene is a key cytosolic enzyme in the arachidonic acid metabolism pathways (FDR=0.025, Table 1). The Alox15 protein and its metabolites play important roles in both physiology and pathology in a variety of different tissues [57]. Interestingly, Alox15 is also involved in the modulation of neuronal function such as in axon pathfinding, in particular at the level of the growth cones [58] and in the neuronal protection and survival after axotomy [59]. The *Robo1* (roundabout 1) gene encodes for a membrane receptor and it is expressed both in non-neural tissue (lung and kidney [60,61]) and, more abundantly, in the nervous system (brain, retina and spinal cord), where it plays a pivotal role in axon pathfinding, particularly during the process of midline crossing in the developing spinal cord and hindbrain [62–67]. Since microarray experiments indicated an increased level of the *Alox15* and *Robo1* mRNA, we asked whether they were actually translated into proteins in spinal cultures; that is, we wondered whether interfacing cells with MWCNT platforms affected also their protein level. Upon Western blot experiments, we found that both Alox15 and Robo1 proteins were expressed in control and CNT spinal cultures (Figure 4A and B, left panels). In Figure 4A (plot), the quantification of the Alox15 protein level (normalized to the actin one) showed a significant 58% increase in CNT cultures (0.52 ± 0.12, n=3) with respect to control ones (0.33 ± 0.13, n=3; P<0.05), indicating that the increased Alox15 mRNA corresponds to a parallel raise in the Alox15 protein amount. The presence of the Alox15 protein was also confirmed by immunofluorescence experiments showing that Alox15 was typically localized in the cytoplasm of both control and CNT neurons (confocal images in Figure 4A, bottom). Interestingly, unlike Alox15, the Robo1 protein level (Western blot in Figure 4B) was unchanged in CNT cultures (0.78 ± 0.11, n=3 in control; 0.76 ± 0.24, n=3 in CNT cultures; measures are normalized to the actin level), despite the mRNA increase revealed by the microarray data. The apparent mismatch between the *Robo1* mRNA and the protein level is not surprising, as transcription and translation are governed by independent mechanisms, often with different time constants, therefore these two processes are not always positively correlated [68,69]. Each mRNA has its own rate of decay and translation, and the post-transcriptional and translational steps are regulated by complex and specific mechanisms [69], including microRNA-mediated regulatory loops [70]. The fact that *Alox15* mRNA and protein levels are both increased in CNT cultures compared to controls indicates an *ongoing* rise in the *Alox15* activity at the time point analyzed (8 days *in vitro*, DIV), likely involved in supporting neuronal survival and in guiding neurite processes [58,59]. Conversely, the observation that the *Robo1* mRNA was increased by the MWCNT substrate, while the Robo1 protein was not, suggests a temporal shift between the *Robo1* and *Alox15* MWCNT substrate induced overexpression. More explicitly, the absence of Robo1 additional protein synthesis might indicate a later, within the maturational process, appearance of the effects mediated by the increased Robo1 (for example, at the level of neurite growth [63–67]), i.e. after the developmental stage analyzed (8 DIV). To better clarify the temporal dynamics of *Alox15* and *Robo1* expression, we quantitatively investigated their expression time course by transcript-specific real time PCR amplification (RT-PCR) (Figure 4C) on both control and MWCNT spinal cultures. Both the *Alox15* and *Robo1* gene profiles of CNT cultures from 4 to 10 DIV displayed a progressive increase, which was statistically significant at 10 DIV (n=3 culture series). Interestingly, *Alox15* expression tends to be higher (although not significantly) in MWCNT cultures already at 4 DIVs, while MWCNT impact on *Robo1* expression appears to be delayed compared to *Alox15*. These results support the temporal shift between *Robo1* and *Alox15* overexpression on MWCNT suggested by Western blot experiments, and confirm and strengthen the microarray data.

**Table 1:** Enrichment gene set analysis on KEGG pathways in CNT cultures compared to controls.

**KEGG pathway**	**FDR**
Toll-like receptor signalling pathway	0.000090
Cytokine-cytokine receptor interaction	0.000860
Ubiquitin mediated proteolysis	0.022951
Arachidonic acid metabolism	0.025426
Antigen processing and presentation	0.028659
Complement and coagulation cascades	0.025475
PPAR signaling pathway	0.031632

FDR, fold discovery rate

It is interesting to note that the products of both genes, *Alox15* and *Robo1*, are involved in neurite pathfinding; we may speculate that the over-activity of these genes, induced by neuronal sensing of MWCNT scaffold, is related to an improved ability of neuritis, during their development/regrowth, in responding to guiding cues. Ultimately, CNT neurons could be more effective in arresting neurite growth upon target recognition. In accordance to this hypothesis we reported their shorter neurite, reminiscent of the principal axons described in cultured neurons isolated from older animals [71], where axonal growth arrest reflected an advanced maturation stage [72,73].

**Figure 5:**
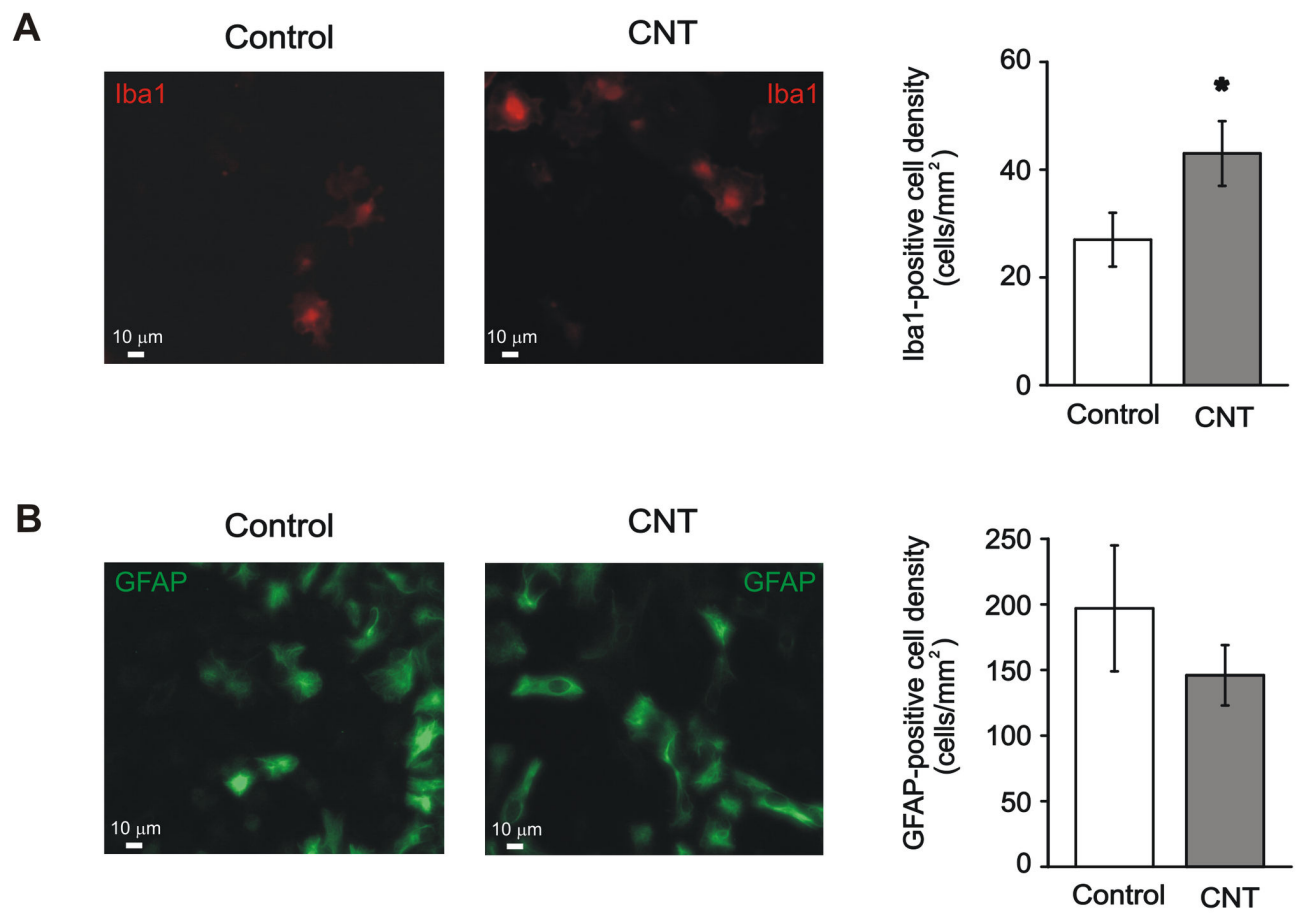
MWCNTs impact on the density of non-neuronal cells in spinal cord cultures. (A) Left, images of control and CNT spinal cultures immunostained for the microglia/macrophage marker Iba1. Right, the density of Iba1-positive cells is increased in CNT cultures (*: P<0.05). (B) Left, images of control and CNT spinal cultures immunostained for the glial marker GFAP. Right, the density of GFAP-positive cells is similar in control and CNT cultures.

An additional issue, emerging from gene analysis, concerns the effects of the MWCNT substrate on non-neuronal cell types. Microarray experiments indicated an increase in the transcription of chemokine-related genes (e.g. *Cxcl10*, *Cxcl11*, *Ccl5*) as well as immune response genes, such as *Irf7*, *Ifit1*, *Ifit3*. Alox15 itself has been recently addressed as implicated in biosynthesis of inflammatory mediators in phagocytes [74,75]. Moreover, by interrogating KEGG pathway database (Kyoto Encyclopedia of Genes and Genomes [42]), we found significant over-representation of pathways involved in immune-related function, such as Toll like Receptor signalling (FDR=0.000090), cytokine-cytokine interaction (p-val 0.00086) and the arachidonic cascade (FDR=0.025), as shown in Table 1. GO enrichment additionally showed also over-represented categories involved in macrophage activation, immune system development and myeloid cell differentiation (Figure 3 and Table S2). Since an increased density of microglial/macrophagic cells on MWCNT could account for this result, we performed immunofluorescence experiments using antibodies against the microglia/macrophage specific marker Iba1 (Ionized calcium binding adaptor molecule 1; Figure 5A [76]) and we found that the density of Iba1-positive cells was significantly increased in CNT cultures (43 ± 6 cells/mm^2^, n=5 cultures) compared to control ones (27 ± 5 cells/mm^2^, n=5 cultures; P<0.05; plot in Figure 5A).

The higher number of Iba-1 positive cells might be related to higher adhesion of this cell category to MWCNT. Alternatively, an increased proliferation, underlying an on-going ignition of reparative or inflammatory processes in the cultured samples, could be involved. Although we cannot exclude the presence of proliferation due to inflammation, several observations argue against this interpretation. In our experiments CNT neurons display a healthy morphology and healthy electrophysiological properties, with no apparent signs of degeneration or even sufferance. In addition, the density of astrocytes, visualized as Glial Fibrillary Acidic Protein (GFAP)-positive cells by immunofluorescence, was the same in control (197 ± 48 cells/mm^2^, n=4 cultures) and on MWCNT (146 ± 23 cells/mm^2^, n=4 cultures; Figure 5B), with no sign of reactive gliosis usually accompanying inflammatory responses [77,78]. Finally, Iba-1 positive cells did not display in CNT cultures a different morphology when compared to controls (control: 88.9% amoeboid morphology, 7.9% round, 1.6% rod-like, 1.6% ramified, n=63 cells; CNT: 85% amoeboid, 8.8% round, 3.7% rod-like, 2.5% ramified, n=80 cells [76,79]), suggesting the absence of a functional switch towards activated microglia. Thus, in view of these observations and of the increasing evidences that microglia can also be involved in central nervous system repair [80], we favour the hypothesis of an improved microglia adhesion instead of proliferation due to a reparative activity on MWCNT.

### Conclusion and physiological relevance

Overall here we described a facilitation of functional maturation of spinal neurons on MWCNT accompanied by a selective modulation of gene expression. Our data do not allow establishing a direct cause effect relationship between the MWCNT, gene expression and neuronal behavior. Our hypothesis is that MWCNT might boost the neuronal adhesion to the substrate, triggering the activation of a cascade of intracellular signaling events mediated by adhesion structures that led to long-term changes in transcriptional regulation, differentiation and survival [3,81]. This hypothesis is supported by the observed adhesion modality of neurons to MWCNTs, displaying typical tight membrane contacts (shown in Figure 1C) that are absent in fibers grown on control substrate (Figure S1 A). Furthermore, by Western blot experiments we observed that the expression of the focal adhesion-associated protein paxillin [82,83] is increased in spinal cultures grown on the MWCNT substrate compared to control (Figure S1 B). Paxillin is involved in focal adhesions-mediated intracellular signaling pathways [82,83], and cell attachment to different substrates is known to selectively modulate gene expression [84]. For example, the adhesion-mediating receptors of the integrin family regulate inactivating K^+^ currents in hippocampal neurons [85], while cadherins mediate the formation of functional, action potential-generating neuronal networks [86]. The strong neuronal adhesion/interaction with MWCNT is therefore likely to recruit similar classes of adhesion molecules and initiate certain cascades of events.

Alternatively, the physical and chemical features together with the topography of MWCNT might improve the reparative ability of the dissociated spinal cells, due to higher density, or activation, of microglia cells and increasing the transcription of molecules involved in neuronal survival after damage [59] and that play important roles in neurite pathfinding [58,63–67].

In this framework, MWCNT scaffolds may possess physical and chemical properties able to improve recovery and promote excitability of dissociated neonatal neurons. On the other hand, MWCNT may trigger microglial reparative processes leading to an accelerated maturation of neurons, mimicking an aging environment.

## Supporting Information

Table S1 **Complete list of differentially expressed genes in CNT cultures compared to control.** Differentially expressed genes (DEGs) analysis identified a total of 5 down-regulated and 46 up-regulated genes in CNT cultures compared to controls (with a fold change (FC) > 2 and FC<0.5, corrected p-value < 0.05.
(DOC)

Table S2 **List of the Gene Ontology biological processes with statistics.**
(DOC)

Figure S1 **Carbon nanotubes boost cell adhesion to the substrate (A) Scanning electron microscope images showing neuronal fibers grown on MWCNTs (top) or on control substrate (bottom).** The tight contacts with the substrate typical of fibers grown on MWCNTs (arrows) are not present on control ones. (B) Western blot analysis of the paxillin protein (migrated at approximately 65 KD) from control (left lane) and CNT (right lane) cultures. Paxillin expression (normalized to actin) is higher on MWCNTs compared to control substrate (0.86 vs 0.42, respectively; one culture series).
(TIF)
