# Supplementary material for: Adhesion to Carbon Nanotube Conductive Scaffolds Forces Action-Potential Appearance in Immature Rat Spinal Neurons
Source: PLoS One. 2013 Aug 12;8(8):e73621. doi: 10.1371/journal.pone.0073621 (PMC3741175; doi:10.1371/journal.pone.0073621)
Supplement: Table S1 — Complete list of differentially expressed genes in CNT cultures compared to control. Differentially expressed genes (DEGs) analysis identified a total of 5 down-regulated and 46 up-regulated genes in CNT cultures compared to controls (with a fold change (FC) > 2 and FC<0.5, corrected p-value < 0.05. (DOC) [file pone.0073621.s001.doc]

| *Up-regulated genes* | | |
| --- | --- | --- |
| *Gene Symbol* | *Description* | *FC* |
| *Ifit3* | interferon-induced protein with tetratricopeptide repeats 3 | 13.03780965 |
| *Slfn3* | schlafen 3 | 7.451564829 |
| *Oas1a* | 2'-5' oligoadenylate synthetase 1° | 7.407407407 |
| *Mx1* | myxovirus (influenza virus) resistance 1 | 7.830853563 |
| *LOC679623* | similar to Nuclear autoantigen Sp-100 (Speckled 100 kDa) (Nuclear dot-associated Sp100 protein) | 6.309148265 |
| *Oasl1* | 2'-5' oligoadenylate synthetase-like 1 | 7.082152975 |
| *Irf7* | interferon regulatory factor 7 | 6.30517024 |
| *G1p2* | interferon, alpha-inducible protein (clone IFI-15K) | 6.887052342 |
| *Rsad2* | radical S-adenosyl methionine domain containing 2 | 7.77000777 |
| *isg12(b)* | putative ISG12(b) protein | 5.361930295 |
| *Zbp1* | Z-DNA binding protein 1 | 5.078720163 |
| *Robo1* | RoBo-1 | 3.518648839 |
| *Ifit1* | interferon-induced protein with tetratricopeptide repeats 1 | 4.723665564 |
| *Mx2* | myxovirus (influenza virus) resistance 2 | 4.62962963 |
| *LOC690768* | hypothetical protein LOC690768 | 4.081632653 |
| *Rtp4* | receptor transporter protein 4 | 4.372540446 |
| *Mmp12* | matrix metallopeptidase 12 | 4.585052728 |
| *Lgals9* | lectin, galactose binding, soluble 9 | 2.864508737 |
| *F10* | coagulation factor X | 3.280839895 |
| *Lyve1* | lymphatic vessel endothelial hyaluronan receptor 1 | 3.086419753 |
| *RT1-S3* | RT1 class Ib, locus S3 | 4.140786749 |
| *Mmp9* | matrix metallopeptidase 9 | 4.4345898 |
| *Sfrp5* | secreted frizzled-related sequence protein 5 | 3.86996904 |
| *Prl6a1* | prolactin family 6, subfamily a, member 1 | 3.320053121 |
| *Oas1i* | 2 ' -5 ' oligoadenylate synthetase 1I | 3.494060098 |
| *Ccr1* | chemokine (C-C motif) receptor 1 | 4.873294347 |
| *Cmpk2* | cytidine monophosphate (UMP-CMP) kinase 2, mitochondrial | 3.731343284 |
| *Dhx58* | DEXH (Asp-Glu-X-His) box polypeptide 58 | 3.824091778 |
| *Cxcl11* | chemokine (C-X-C motif) ligand 11 | 3.103662322 |
| *Dnase1l3* | deoxyribonuclease 1-like 3 | 4.574565416 |
| *Slc6a12* | solute carrier family 6 (neurotransmitter transporter, betaine/GABA), member 12 | 2.745744097 |
| *Alox15* | arachidonate 15-lipoxygenase | 2.781641168 |
| *Fabp4* | fatty acid binding protein 4, adipocyte | 4.582951421 |
| *Cxcl10* | chemokine (C-X-C motif) ligand 10 | 3.513703443 |
| *Ube1l* | ubiquitin-activating enzyme E1-like | 2.610966057 |
| *Slc16a3* | solute carrier family 16 (monocarboxylic acid transporters), member 3 | 2.751031637 |
| *Slco4a1* | solute carrier organic anion transporter family, member 4a1 | 2.407318247 |
| *Apol9a* | apolipoprotein L 9a | 2.591344908 |
| *Ifi204* | interferon activated gene 204 | 2.986857826 |
| *Ly6e* | lymphocyte antigen 6 complex, locus E | 2.1602938 |
| *Bst2* | bone marrow stromal cell antigen 2 | 2.596053998 |
| *Ccl5* | chemokine (C-C motif) ligand 5 | 2.326663564 |
| *Slc28a2* | solute carrier family 28 (sodium-coupled nucleoside transporter), member 2 | 3.792188093 |
| *RGD1562323* | similar to fatty acid translocase/CD36 | 3.756574005 |
| *Ddx60* | DEAD (Asp-Glu-Ala-Asp) box polypeptide 60 | 3.151591554 |
| *Ela1* | elastase 1, pancreatic | 3.531073446 |
|  | | |
| *Downregulated genes* | | |
| *Gene Symbol* | *Description* | *FC* |
| *Shisa3* | shisa homolog 3 (Xenopus laevis) | 0.28458408 |
| *Aspn* | asporin | 0.279189234 |
| *Nedd4* | neural precursor cell expressed, developmentally down-regulated gene 4 | 0.328731098 |
| *Gas2* | growth arrest-specific 2 | 0.351790614 |
| *Fbln2* | fibulin 2 | 0.366999413 |

**Table S1**. Complete list of differentially expressed genes in CNT cultures compared to control. Differentially expressed genes (DEGs) analysis identified a total of 5 down-regulated and 46 up-regulated genes in CNT cultures compared to controls (with a fold change (FC)>2 and FC<0.5, corrected p-value<0.05.
