# Supplementary material for: Adhesion to Carbon Nanotube Conductive Scaffolds Forces Action-Potential Appearance in Immature Rat Spinal Neurons
Source: PLoS One. 2013 Aug 12;8(8):e73621. doi: 10.1371/journal.pone.0073621 (PMC3741175; doi:10.1371/journal.pone.0073621)
Supplement: Table S2 — List of the Gene Ontology biological processes with statistics. (DOC) [file pone.0073621.s002.doc]

| **Category** | **FDR** |
| --- | --- |
| GO:0045794~negative regulation of cell volume | 0.000000 |
| GO:0009615~response to virus | 0.000020 |
| GO:0000270~peptidoglycan metabolic process | 0.000595 |
| GO:0002687~positive regulation of leukocyte migration | 0.000651 |
| GO:0002685~regulation of leukocyte migration | 0.001558 |
| GO:0051272~positive regulation of cell motility | 0.001993 |
| GO:0040017~positive regulation of locomotion | 0.002326 |
| GO:0045351~interferon type I biosynthetic process | 0.003228 |
| GO:0032963~collagen metabolic process | 0.005406 |
| GO:0044254~multicellular organismal protein catabolic process | 0.005744 |
| GO:0006935~chemotaxis | 0.005792 |
| GO:0015858~nucleoside transport | 0.005991 |
| GO:0050900~leukocyte migration | 0.006028 |
| GO:0043450~alkene biosynthetic process | 0.006029 |
| GO:0044266~multicellular organismal macromolecule catabolic process | 0.006127 |
| GO:0042330~taxis | 0.006274 |
| GO:0030307~positive regulation of cell growth | 0.006364 |
| GO:0042403~thyroid hormone metabolic process | 0.006517 |
| GO:0044268~multicellular organismal protein metabolic process | 0.006564 |
| GO:0006921~cell structure disassembly during apoptosis | 0.006648 |
| GO:0045793~positive regulation of cell size | 0.006981 |
| GO:0030334~regulation of cell migration | 0.008289 |
| GO:0006801~superoxide metabolic process | 0.008560 |
| GO:0006308~DNA catabolic process | 0.008809 |
| GO:0031349~positive regulation of defense response | 0.008949 |
| GO:0008284~positive regulation of cell proliferation | 0.008981 |
| GO:0006691~leukotriene metabolic process | 0.009760 |
| GO:0051270~regulation of cell motility | 0.010301 |
| GO:0042116~macrophage activation | 0.010356 |
| GO:0002474~antigen processing and presentation of peptide antigen via MHC class I | 0.011313 |
| GO:0045927~positive regulation of growth | 0.016715 |
| GO:0006954~inflammatory response | 0.017700 |
| GO:0006997~nuclear organization and biogenesis | 0.017735 |
| GO:0030099~myeloid cell differentiation | 0.018017 |
| GO:0030097~hemopoiesis | 0.018103 |
| GO:0030595~leukocyte chemotaxis | 0.018757 |
| GO:0048534~hemopoietic or lymphoid organ development | 0.020252 |
| GO:0002520~immune system development | 0.022418 |
| GO:0002521~leukocyte differentiation | 0.023217 |
| GO:0006022~aminoglycan metabolic process | 0.023319 |
| GO:0030278~regulation of ossification | 0.023781 |
| GO:0050727~regulation of inflammatory response | 0.023887 |
| GO:0050678~regulation of epithelial cell proliferation | 0.024334 |
| GO:0031347~regulation of defense response | 0.024485 |
| GO:0043285~biopolymer catabolic process | 0.024563 |
| GO:0001503~ossification | 0.026719 |
| GO:0031214~biomineral formation | 0.027299 |
| GO:0002274~myeloid leukocyte activation | 0.027799 |
| GO:0015931~nucleobase, nucleoside, nucleotide and nucleic acid transport | 0.031415 |
| GO:0009306~protein secretion | 0.032056 |
| GO:0046850~regulation of bone remodeling | 0.033419 |
| GO:0030198~extracellular matrix organization and biogenesis | 0.034075 |
| GO:0042089~cytokine biosynthetic process | 0.044906 |
| GO:0002449~lymphocyte mediated immunity | 0.047045 |
| GO:0006836~neurotransmitter transport | 0.047211 |
| GO:0016049~cell growth | 0.047350 |
| GO:0006633~fatty acid biosynthetic process | 0.047885 |
| GO:0006869~lipid transport | 0.048025 |
| GO:0009617~response to bacterium | 0.048755 |
| GO:0016477~cell migration | 0.048815 |

**Table S2**. Categories of the biological processes of gene ontology with statistics.
