# Supplementary figures and images for: Adhesion to Carbon Nanotube Conductive Scaffolds Forces Action-Potential Appearance in Immature Rat Spinal Neurons
Source: PLoS One. 2013 Aug 12;8(8):e73621. doi: 10.1371/journal.pone.0073621 (PMC3741175; doi:10.1371/journal.pone.0073621)

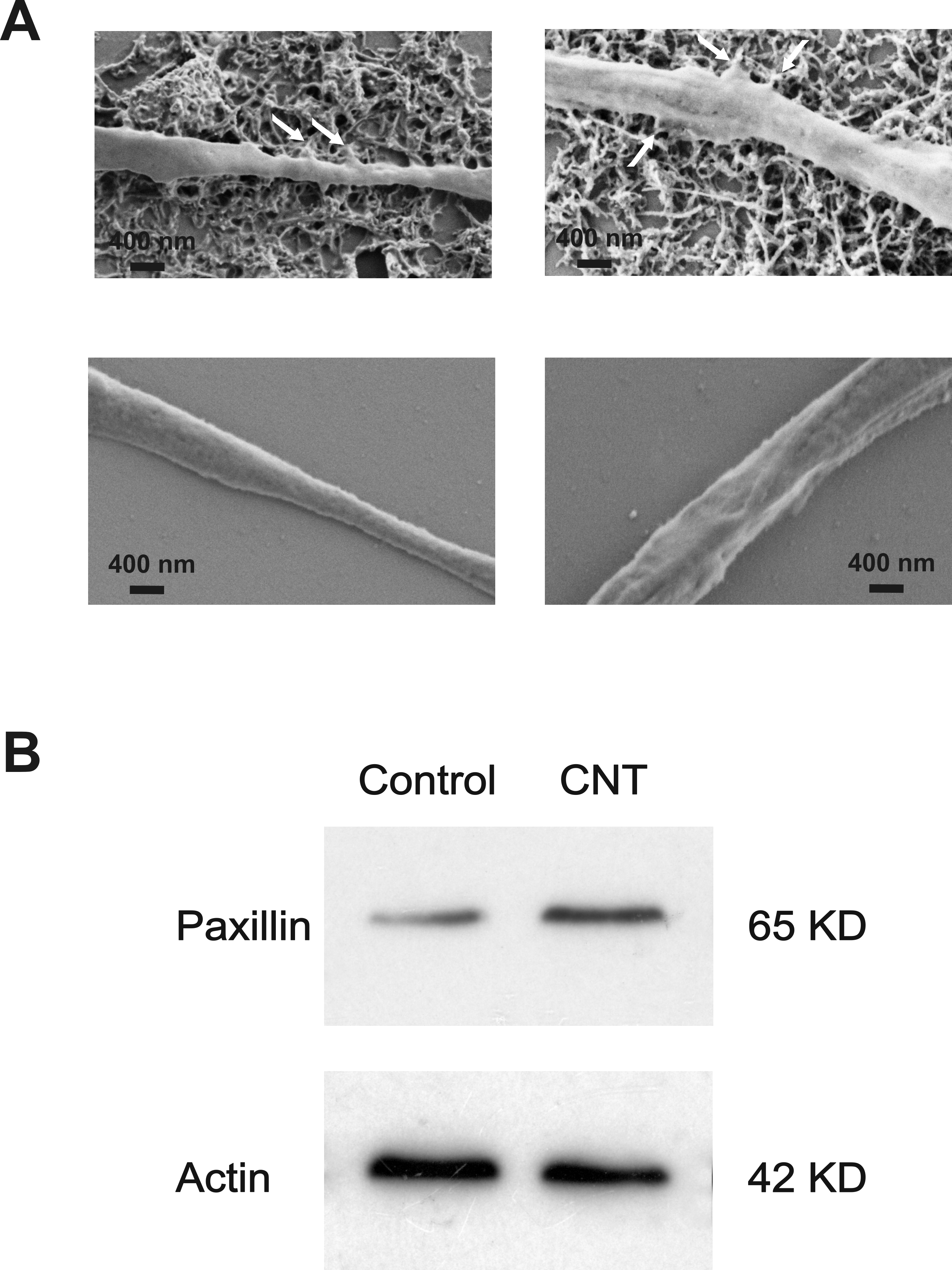

Supplement: Figure S1 — Carbon nanotubes boost cell adhesion to the substrate (A) Scanning electron microscope images showing neuronal fibers grown on MWCNTs (top) or on control substrate (bottom). The tight contacts with the substrate typical of fibers grown on MWCNTs (arrows) are not present on control ones. (B) Western blot analysis of the paxillin protein (migrated at approximately 65 KD) from control (left lane) and CNT (right lane) cultures. Paxillin expression (normalized to actin) is higher on MWCNTs compared to control substrate (0.86 vs 0.42, respectively; one culture series). (TIF) [file pone.0073621.s003.tif]
